# Echocardiography-Unmasked Pulmonary Hypertension in Acute Respiratory Distress Syndrome (ARDS): A Fatal Case of Biventricular Failure

**DOI:** 10.7759/cureus.90366

**Published:** 2025-08-18

**Authors:** Mohamed M Dar, Ihab Nagla, Ramy Kishk

**Affiliations:** 1 Critical Care Medicine Department, Imperial College Healthcare National Health Service (NHS) Trust, London, GBR; 2 Critical Care Department, Imperial College Healthcare National Health Service (NHS) Trust, London, GBR; 3 Critical Care Department, Faculty of Medicine, Cairo University, Cairo, EGY

**Keywords:** acute respiratory distress syndrome, biventricular failure, pulmonary hypertension, right ventricular dysfunction, transthoracic echocardiography

## Abstract

We report a case of a 65-year-old hypertensive male patient admitted to the ICU with altered consciousness, shock, and profound hypoxemia. He met the Berlin criteria for severe acute respiratory distress syndrome (ARDS) and was intubated immediately. Despite mechanical ventilation and vasopressor support (norepinephrine 3.5 µg/kg/min), oxygenation remained critically impaired (partial pressure of arterial oxygen/fraction of inspired oxygen (PaO₂/FiO₂) ratio: 50 mmHg). Early bedside transthoracic echocardiography revealed severe pulmonary hypertension (PH) (pulmonary artery systolic pressure (PASP): 85 mmHg), reduced right ventricular function (tricuspid annular plane systolic excursion (TAPSE): 1.5 cm), and left ventricular systolic dysfunction (left ventricular ejection fraction (LVEF): 30%), indicating acute cor pulmonale and biventricular failure. The patient’s course was marked by refractory multiorgan failure, including acute renal injury and neurologic unresponsiveness. Despite full supportive measures, he died within 30 days of admission. This case emphasizes the prognostic value of early echocardiographic assessment in ARDS. Identifying elevated pulmonary pressures and biventricular dysfunction early may aid in risk stratification, guide management, and potentially improve outcomes in critically ill patients.

## Introduction

Acute respiratory distress syndrome (ARDS) is a critical condition characterized by diffuse alveolar damage, impaired gas exchange, and severe hypoxemia. Despite adherence to evidence-based interventions such as lung-protective ventilation, neuromuscular blockade, and prone positioning, ARDS continues to carry a high mortality rate, particularly in patients who progress to multiorgan failure [1,2]. Emerging data underscore the importance of right ventricular (RV) dysfunction and pulmonary hypertension (PH) in the pathophysiology and clinical trajectory of ARDS [3,4]. PH, defined as a mean pulmonary artery pressure >25 mmHg, often develops due to hypoxic pulmonary vasoconstriction, elevated intrathoracic pressures, and pulmonary vascular remodeling. When severe, PH can precipitate acute RV strain, a phenomenon known as acute cor pulmonale, which may evolve into biventricular failure in critically ill patients [5]. Although PH and ventricular dysfunction may not always be modifiable in advanced stages of illness, their early recognition has growing prognostic relevance. Bedside transthoracic echocardiography (TTE) provides a rapid, non-invasive method to assess pulmonary pressures and cardiac function in unstable patients. Parameters such as tricuspid regurgitation peak gradient (TR PG), tricuspid annular plane systolic excursion (TAPSE), and left ventricular ejection fraction (LVEF) can offer valuable insights into cardiopulmonary interactions, especially when standard clinical signs are obscured by sedation or mechanical ventilation [6]. This case report describes a patient with fulminant ARDS who developed severe PH and biventricular failure early in the ICU course. It highlights how early echocardiographic findings may serve as prognostic indicators of an unfavorable trajectory, supporting their integration into risk stratification and decision-making in critically ill patients with ARDS.

## Case presentation

Initial presentation and admission

The patient, a 65-year-old male patient with a known history of hypertension, was brought to the emergency department after experiencing progressive shortness of breath, reduced urine output, and altered mental status over the preceding 48 hours. According to his family, he had become increasingly lethargic, hypoxic, and dyspneic, culminating in unresponsiveness on the day of admission. There was no history of chest pain, palpitations, fever, or focal neurological deficits.

On arrival at the ICU, he was comatose with a Glasgow Coma Scale (GCS) score of 3/15, requiring immediate endotracheal intubation and initiation of invasive mechanical ventilation. On initial assessment, he was tachycardic (150 bpm), hypotensive with a mean arterial pressure (MAP) of 40 mmHg, and tachypneic at 26 breaths per minute. Oxygen saturation was 78% on high-flow oxygen. Chest auscultation revealed diffuse bilateral crackles. Cardiac examination revealed normal heart sounds without murmurs or gallops. There was no jugular venous distension, peripheral edema, or organomegaly. The abdomen was soft and non-tender. Extremities were cool and mottled, consistent with peripheral hypoperfusion. There were no signs of focal neurologic deficits or raised intracranial pressure. The arterial blood gas (ABG) analysis performed on admission revealed: pH, 6.70; PaO₂, 52 mmHg; PaCO₂, 88 mmHg; HCO₃⁻, 11 mmol/L; base excess, 18 mmol/L; and SaO₂, 78%.

Given the constellation of profound hypoxemia, hypotension, and decreased consciousness, the initial differential diagnosis included septic shock with pneumonia, massive pulmonary embolism, acute coronary syndrome, cardiogenic shock, and acute decompensated heart failure. A chest radiograph showed bilateral diffuse infiltrates without focal consolidation or cardiomegaly. ECG revealed sinus tachycardia with no ischemic changes, and troponin levels were slightly elevated but not rising. The absence of focal findings and the presence of diffuse pulmonary infiltrates, along with the ratio of partial pressure of arterial oxygen/fraction of inspired oxygen (PaO₂/FiO₂) of 50 mmHg, fulfilled the Berlin criteria for severe ARDS.

Laboratory investigations revealed severe mixed respiratory and metabolic acidosis on arterial blood gas (pH 6.7), elevated serum creatinine at 3.8 mg/dL, indicating acute kidney injury, hyponatremia (Na⁺ of 127 meq/L), hyperkalemia (K⁺ of 5.6 meq/L), anemia (hematocrit at 27%), and marked leukocytosis (white blood cells (WBC) at 20 × 10⁹/L). The patient was classified as being in shock, with vasopressor support initiated using high-dose norepinephrine (3.5 µg/kg/min) (Table 1).

**Table 1:** Clinical, laboratory, and echo parameters of the patient FiO₂: Fraction of Inspired Oxygen; PaO₂: Partial Pressure of Arterial Oxygen; TR: Tricuspid Regurgitation; PG: Pressure Gradient; RAP: Right Atrial Pressure; PASP: Pulmonary Artery Systolic Pressure; TAPSE: Tricuspid Annular Plane Systolic Excursion; LVEF: Left Ventricular Ejection Fraction; E/E’: Ratio of Early Mitral Inflow Velocity to Mitral Annular Early Diastolic Velocity; RV: Right Ventricle; LV: Left Ventricle.

Parameter	Value	Reference Range
Clinical Parameters	-	-
Mean Arterial Pressure (mmHg)	40 mmHg	70–100 mmHg
Heart Rate (bpm)	150 bpm	60–100 bpm
Respiratory Rate (cycles/min)	26/min	12–20/min
Temperature (°C)	37.4°C	36.1–37.2°C
Glasgow Coma Scale	3	15
FiO₂ (%)	>50%	<50%
PaO₂/FiO₂ Ratio	50 mmHg	>300 mmHg
Amount of Fluid Received (last 6 hrs, mL)	2000	-
Vasopressor Use During Echo	Yes	No
Norepinephrine Equivalent (µg/kg/min)	3.5 µg/kg/min	<0.1 µg/kg/min
Mechanically Ventilated During Echo	Yes	No
Laboratory Findings	-	-
pH	6.7	7.35–7.45
Sodium (mmol/L)	127 mmol/L	135–145 mmol/L
Potassium (mmol/L)	5.6 mmol/L	3.5–5.1 mmol/L
Creatinine (mg/dL)	3.8 mg/dL	0.6–1.2 mg/dL
Acute Renal Failure	Yes	No
Hematocrit (%)	27%	38–50% (male)
White Blood Cell Count (×10⁹/L)	20×10⁹/L	4–11×10⁹/L
Echocardiographic Findings	-	-
TR max PG (mmHg)	70 mmHg	<30 mmHg
RAP (mmHg)	15 mmHg	3–10 mmHg
PASP (mmHg)	85 mmHg	<35 mmHg
TAPSE (mm)	15 mm	≥17 mm
RV Dysfunction	Yes	No
LVEF (%)	30%	≥55%
LV Systolic Dysfunction	Yes	No
LV E/E’	13.2	<14
LV Diastolic Dysfunction	Yes	No

Progressive deterioration and echocardiographic evaluation

Despite fluid resuscitation with 2000 mL of intravenous crystalloids over the initial 6 hours, the patient remained profoundly hypotensive with escalating oxygen requirements. FiO₂ was maintained above 50% with no meaningful improvement in oxygenation. The PaO₂/FiO₂ ratio remained at 50 mmHg, confirming the diagnosis of severe ARDS.

**Figure 1:**
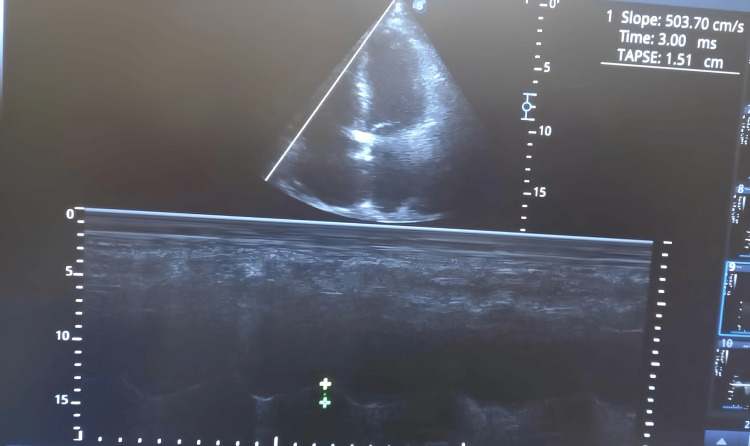
M-mode echocardiographic measurement of Tricuspid Annular Plane Systolic Excursion (TAPSE) from the apical four-chamber view Tricuspid Annular Plane Systolic Excursion (TAPSE), mm: millimeter, ms: millisecond.

Due to persistent shock, worsening hypoxemia, and evidence of multisystem involvement - including acute kidney injury and neurologic unresponsiveness - bedside TTE was performed early during ICU admission to assess cardiac function and clarify hemodynamic status. The study revealed a severely elevated pulmonary artery systolic pressure (PASP) of 85 mmHg, derived from a tricuspid regurgitation (TR) peak gradient of 70 mmHg and an estimated right atrial pressure (RAP) of 15 mmHg. The echocardiogram also demonstrated significant right ventricular (RV) dysfunction, with a TAPSE of 1.5 cm and dilated RV dimensions, consistent with acute cor pulmonale in the context of pulmonary hypertension (PH) (Figure 1).

In addition, biventricular impairment was evident. The left ventricular ejection fraction (LVEF) was reduced to 30%, indicating global systolic dysfunction. The E/E′ ratio was 13.2, suggestive of elevated left ventricular (LV) filling pressures and diastolic dysfunction. These findings collectively confirmed the presence of severe PH with associated biventricular failure in the setting of ARDS (Table 1).

These findings pointed to acute cor pulmonale in the context of ARDS-induced PH, compounded by left ventricular dysfunction. Notably, the echocardiogram was performed while the patient was mechanically ventilated and on vasopressor support, emphasizing the critical severity of his condition and the utility of echocardiography for early risk assessment.

Clinical course and outcome

In the days following admission, the patient’s clinical status remained grave. He was fully sedated and mechanically ventilated on high FiO₂ and positive end-expiratory pressure (PEEP), yet oxygenation remained critically impaired. Renal function worsened, with near-anuric output despite adequate fluid resuscitation and vasopressor support. A nephrology consultation diagnosed acute tubular necrosis secondary to shock and sepsis. Continuous renal replacement therapy (CRRT) was considered but deferred due to persistent hypotension and limited hemodynamic reserve.

Cardiovascular support was escalated with the addition of vasopressin, and empiric broad-spectrum antibiotics were initiated to cover suspected superimposed pneumonia and sepsis. Despite full ICU support, there was no meaningful neurological improvement. Sedation holidays revealed no response beyond preserved brainstem reflexes, and neurology consultation suggested a guarded prognosis.

In the second week of admission, serial echocardiography continued to show persistent RV dysfunction and unchanged PASP. Lung compliance worsened, and radiological imaging demonstrated bilateral consolidation with evolving fibrosis. Repeated ABG analyses showed continued hypoxemia and severe metabolic acidosis, requiring escalating ventilatory settings, including recruitment maneuvers and prone positioning, with only transient improvements.

By the third week, the patient remained ventilator-dependent, anuric, and neurologically unresponsive. Hematologic parameters showed rising inflammatory markers, persistent leukocytosis, and thrombocytopenia. Antimicrobial therapy was adjusted based on sputum culture results for identifying multidrug-resistant organisms.

Despite maximal supportive care and multidisciplinary efforts, the patient never achieved hemodynamic stability or ventilator weaning. CRRT could not be safely initiated due to persistent vasoplegia. Progressive right heart failure, compounded by left ventricular dysfunction and unresolving ARDS, culminated in multiorgan failure. The patient did not survive beyond the acute phase of illness, experienced cardiac arrest, could not be resuscitated, and was declared deceased within 30 days of ICU admission. He experienced zero ventilator-free days, reflecting the fulminant and non-recoverable trajectory of disease.

## Discussion

The clinical trajectory of this patient exemplifies the prognostic implications of PH and biventricular dysfunction in the setting of severe ARDS. Despite adherence to established ARDS management protocols [7] - including early mechanical ventilation and vasopressor support - the patient experienced rapid clinical deterioration, marked by refractory hypoxemia, severe acidosis, and progressive multi-organ failure. Early bedside TTE uncovered essential information regarding the underlying cardiopulmonary pathophysiology, with elevated PASP, impaired RV systolic function, and reduced LVEF. These findings, in conjunction with severe ARDS, predicted a fatal trajectory. PH in ARDS is often multifactorial, stemming from hypoxic pulmonary vasoconstriction, endothelial injury, inflammatory mediator release, and elevated intrathoracic pressures from mechanical ventilation. In such settings, acute RV afterload increases may precipitate RV dilation and dysfunction, a phenomenon referred to as acute cor pulmonale. In our patient, a PASP of 85 mmHg, combined with a TAPSE of 1.5 cm and elevated RAP, indicated significant RV strain. These findings are consistent with previous studies [8, 9] reporting that elevated PASP and reduced TAPSE are independent predictors of mortality in ARDS patients with PH.

Importantly, our case also demonstrated concurrent LVEF (30%) and an elevated E/E′ ratio (13.2), indicating elevated LV filling pressures and diastolic dysfunction. While LVEF is often preserved in ARDS patients, the presence of biventricular failure in this case may reflect systemic inflammation, sepsis-induced myocardial depression, and possible underlying ischemic or stress cardiomyopathy. The co-existence of RV and LV dysfunction substantially compromises hemodynamics and oxygen delivery, reducing the reserve to tolerate ventilator-associated pressures and fluid shifts. The echocardiographic findings in our patient align with data from observational cohorts [10,11], which have consistently shown that elevated PASP, low TAPSE, high RAP, and impaired LVEF are strongly associated with increased 30-day mortality and prolonged ventilator dependence in ARDS with PH. In particular, TAPSE ≤1.5 cm and PASP >56 mmHg have been proposed as critical cutoffs for risk stratification [12]. These values were exceeded in our patient early in the ICU course, emphasizing the utility of echocardiography for early prognostication. Despite aggressive resuscitative measures, including vasopressor escalation and ventilatory optimization, our patient demonstrated no improvement in oxygenation or hemodynamics. CRRT was deferred due to persistent vasoplegia, reflecting a clinical paradox: while renal support may offer metabolic stabilization, its initiation often requires hemodynamic reserve that critically ill patients lack. The presence of severe PH further complicates volume management, where fluid resuscitation may worsen RV failure, yet diuresis or ultrafiltration is often hemodynamically intolerable.

Neurologic non-responsiveness and rising inflammatory markers indicated irreversible multi-organ dysfunction. Serial echocardiograms continued to show persistent biventricular impairment and severe PH, reflecting a static or worsening cardiopulmonary profile. The trajectory of this case supports recent findings [13] that RV dysfunction in ARDS is not merely a marker of disease severity but a direct contributor to adverse outcomes, including death. Overall, this case underscores the need for early and comprehensive cardiac assessment in ARDS patients. While guidelines recommend echocardiographic evaluation in shock or unexplained hypoxemia, its prognostic integration remains inconsistent in clinical practice. As demonstrated here, echocardiography can unmask silent cardiopulmonary interactions, particularly in patients with evolving or refractory respiratory failure. Future studies are warranted to refine the timing, frequency, and prognostic value of echocardiographic parameters in ARDS, and to determine whether tailored interventions based on early echocardiographic findings - such as early vasodilator therapy, RV protective ventilation, or preload optimization - can improve outcomes in this high-risk population.

## Conclusions

In severe ARDS, early bedside echocardiography can unmask pulmonary hypertension and biventricular dysfunction, offering critical prognostic insight. Identifying these abnormalities early may guide risk stratification, optimize supportive strategies, and inform multidisciplinary decision-making. This case highlights that when such cardiopulmonary compromise coexists with multiorgan failure, the prognosis is extremely poor despite maximal therapy. Incorporating early echocardiographic assessment into standard ARDS evaluation could improve clinical awareness and facilitate timely, targeted interventions.
